# Alterations of the intrinsic amygdala‐hippocampal network in juvenile myoclonic epilepsy

**DOI:** 10.1002/brb3.2274

**Published:** 2021-07-05

**Authors:** Dong Ah Lee, Junghae Ko, Ho‐Joon Lee, Hyung Chan Kim, Bong Soo Park, Sihyung Park, Il Hwan Kim, Jin Han Park, Yoo Jin Lee, Kang Min Park

**Affiliations:** ^1^ Department of Neurology Haeundae Paik Hospital Inje University College of Medicine Busan Republic of Korea; ^2^ Department of Internal Medicine Haeundae Paik Hospital Inje University College of Medicine Busan Republic of Korea; ^3^ Department of Radiology Haeundae Paik Hospital Inje University College of Medicine Busan Republic of Korea

**Keywords:** amygdala, hippocampus, juvenile myoclonic epilepsy

## Abstract

**Introduction:**

Several lines of evidence suggest that the amygdala–hippocampus is involved in the epileptogenic network of juvenile myoclonic epilepsy (JME). The aim of this study was to investigate the alterations in the individual nuclei of the amygdala and hippocampal subfields, and the intrinsic amygdala‐hippocampal network of patients with JME compared to healthy controls.

**Methods:**

This retrospective study conducted at a single tertiary hospital involved 35 patients with newly diagnosed JME, and 34 healthy subjects. We calculated the individual structural volumes of 18 nuclei in the amygdala, and 38 hippocampal subfields using three‐dimensional volumetric T1‐weighted imaging and FreeSurfer program. We also performed an analysis of the intrinsic amygdala‐hippocampal global and local network based on these volumes using a graph theory and Brain Analysis using Graph Theory (BRAPH) program. We investigated the differences in these volumes and network measures between patients with JME and healthy controls.

**Results:**

There were no significant volume differences in the nuclei of the amygdala and hippocampal subfields between patients with JME and healthy controls. However, we found significant differences in the global network between patients with JME and healthy controls. The mean clustering coefficient was significantly decreased in patients with JME compared to healthy controls (0.473 vs. 0.653, *p *= .047). In addition, specific regions in the hippocampal subfields showed significant differences in the local network between the two groups. The betweenness centrality of the right CA1‐head, right hippocampus–amygdala‐transition area, left hippocampal fissure, left fimbria, and left CA3‐head, was increased in patients with JME compared to healthy controls.

**Conclusion:**

The intrinsic amygdala‐hippocampal global and local networks differed in patients with JME compared to healthy controls, which may be related to the pathogenesis of JME, and memory dysfunction in patients with JME.

## Graphical abstract

We investigate the alterations in the individual nuclei volume of amygdala and hippocampal subfields in JME. We also analyze intrinsic amygdala‐hippocampal network in JME. There are no volume differences in the nuclei of the amygdala and hippocampal subfields but significant differences in the global and local networks between patients with JME and healthy controls exist.

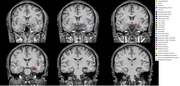

## 1 INTRODUCTION

Juvenile myoclonic epilepsy (JME) is the most common genetic generalized epilepsy associated with various seizure types such as myoclonic, generalized tonic–clonic, and absence seizures (Baykan & Wolf, 2017; Wolf et al., 2015). Visual inspection of the routine brain magnetic resonance imaging (MRI) is normal in patients with JME. However, recent advances in computational analysis have revealed focal abnormalities in the cortical and subcortical gray matter, particularly the thalamus and frontal cortex (Kim, 2017). Furthermore, studies have suggested significant alterations in the brain network of patients with JME using various modalities, such as electroencephalography (EEG) (Kim et al., 2020; Lee & Park, 2019 ), structural MRI (Lee et al., 2020; Park et al., 2017), functional MRI (Kim et al., 2014; Kim et al., 2019), and magnetoencephalography (MEG) (Krzeminski et al., 2020; Routley et al., 2020). The pathophysiology of JME has yet to be elucidated. However, recent studies focused on a systemic disorder of the brain associated with ictogenesis in distributed, bilateral networks involving primarily the thalamus and selective areas of neocortex in patients with JME (Baykan & Wolf, 2017; Wolf et al., 2015).

The amygdala and the hippocampus, juxtaposed in the anterior part of the medial temporal lobe, are key components of the limbic system that play a pivotal role in emotion, learning, and memory (McDonald & Mott, 2017). The amygdala has an essential role in the expression of emotions and the formation of emotion‐related memories, while the hippocampus is involved in the storage of declarative and episodic memory (Bombardi & Di Giovanni, 2013). The amygdala is located immediately in front of the hippocampus, and the interconnections between the amygdala and hippocampus are robust and complex (McDonald & Mott, 2017). The amygdala is composed of heterogeneous nuclei, defined by distinct cytoarchitecture, neurotransmitters, and connectivity patterns. The hippocampus also consists of a number of distinct subfields along the transverse axis. Structural neuroimaging techniques can be used to measure the volumes of the amygdala and hippocampal structures, and FreeSurfer is one of the most widely used programs among the automated tools available. Recently, a new atlas for the segmentation of the 9 nuclei of the amygdala and the 19 hippocampal subfields has been released, which facilitates the simultaneous segmentation of both structures (Iglesias et al., 2015; Saygin et al., 2017). Furthermore, the reliability of the automated segmentation of the nuclei in the amygdala and hippocampal subfields has already been established (Quattrini et al., 2020).

A few studies have elucidated the abnormal structures of the hippocampus as a whole and memory dysfunction in patients with JME (Caciagli et al., 2019; Lin et al., 2013; Ristic et al., 2011). However, no studies have investigated the changes in the individual nuclei of the amygdala and the hippocampal subfields, or the intrinsic amygdala‐hippocampal network based on volumes using a graph theory in patients with JME. The aim of this study was to investigate the structural volume alterations in the individual nuclei of the amygdala and the hippocampal subfields, and the intrinsic amygdala‐hippocampal network in patients with JME compared to healthy controls using a graph theory and the FreeSurfer program. We hypothesized that the intrinsic amygdala‐hippocampal network in patients with JME was different from that of healthy controls.

## 2 METHODS

### 2.1 Subjects

This was a retrospective study conducted in a single tertiary hospital. The study was approved by the Institutional Review Board of our hospital. We enrolled 35 patients with epilepsy who met the following inclusion criteria: (1) a clinical diagnosis of JME based on seizure semiology and EEG findings (Scheffer et al., 2017), (2) newly diagnosed epilepsy with drug‐naive status, (3) normal brain MRI with visual inspection, (4) three‐dimensional T1‐weighted imaging with a 3.0T MRI scanner from January 2015 to July 2020, and (5) normal neurological findings. We investigated the demographic and clinical characteristics of the patients with JME, such as age at the time of MRI, sex, age of seizure onset, duration between the first seizure and the time of brain MRI taken (duration of epilepsy), and seizure types.

We also enrolled 34 age‐ and sex‐matched healthy controls with no past history of medical or neurological diseases. All of the controls also had a normal brain MRI with visual inspections.

### 2.2 Analysis of the volumes in the nuclei of the amygdala and the hippocampal subfields

**FIGURE 1:**
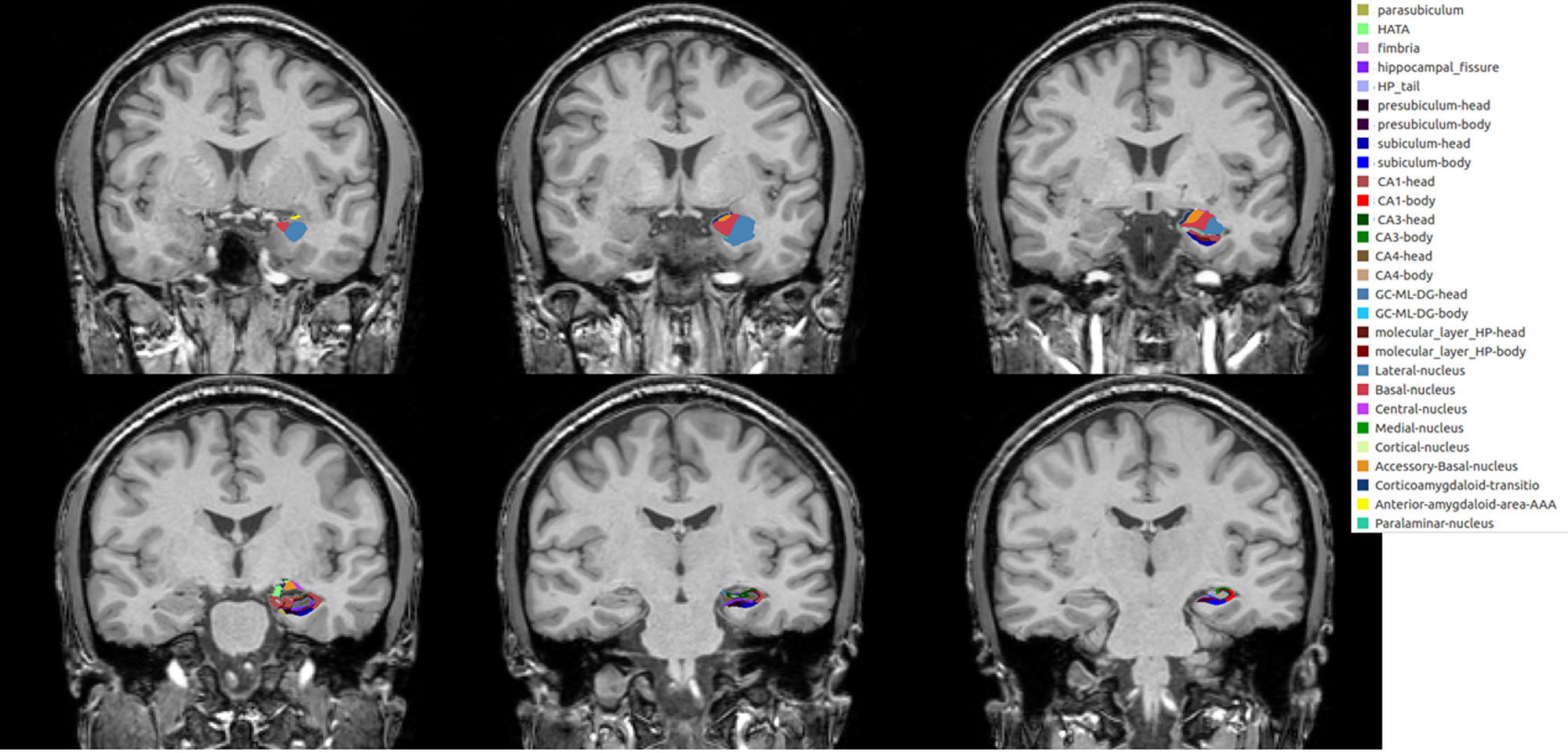
Example of nuclear segmentation in the amygdala and the hippocampal subfields. It shows segmentation on the left side in one of the patients with juvenile myoclonic epilepsy. All segmentations are not shown

All MRI scans in patients with epilepsy and healthy controls were performed using a 3.0 T MRI scanner (AchievaTx, Phillips Healthcare, Best, The Netherlands) equipped with a 32‐channel head coil. All subjects underwent contiguous three‐dimensional volumetric T1‐weighted imaging with a high sagittal resolution appropriate for the analysis of structural volume. The three‐dimensional T1‐weighted images were obtained using a turbo‐field echo sequence with the following parameters: TI = 1300 ms, TR/TE = 8.6/3.96 ms, flip angle = 8°, and 1 mm^3^ isotropic voxel size. Volumetric analysis was performed using the “recon‐all” function in the FreeSurfer program. Then, the individual absolute structural volumes of the 18 nuclei of the amygdala (right and left anterior amygdala area, cortico‐amygdaloid transition area, lateral nucleus, basal nucleus, paralaminar nucleus, accessory basal nucleus, medial nucleus, central nucleus, and cortical nucleus) and the 38 hippocampal subfields (right and left parasubiculum, presubiculum‐head, subiculum‐head, CA1‐head, CA3‐head, CA4‐head, granule cell layer of dentate gyrus‐head, molecular layer‐head, hippocampus–amygdala‐transition area, presubiculum‐body, subiculum‐body, CA1‐body, CA3‐body, CA4‐body, granule cell layer of dentate gyrus‐body, molecular layer‐body, fimbria, hippocampal tail, and hippocampal fissure) were further obtained using the “segmentHA_T1” command (Figure 1). Next, we calculated the volumetric measures using the following equation: The structural volumes (%) = (Absolute structural volumes/Total intracranial volumes) × 100.

### 2.3 Analysis of the intrinsic amygdala‐hippocampal global and local networks

We analyzed the intrinsic amygdala‐hippocampal global and local networks based on volume using graph theory and Brain Analysis using Graph Theory (BRAPH; http://braph.org) (Mijalkov et al., 2017). They were built for each group as a collection of nodes representing brain regions (individual volumes of the nuclei in the amygdala and the hippocampal subfields) connected by edges corresponding to the connections between them. We used the volumes of the 18 nuclei of the amygdala and the 38 hippocampal subfields. The edges were calculated as partial correlation coefficients between every pair of brain regions while controlling for the effects of age and sex. A weighted, undirected connectivity matrix was built for each group. To determine the differences between groups in the intrinsic global amygdala‐hippocampal network, we calculated the average degree, average strength, radius, diameter, eccentricity, characteristic path length, global efficiency, local efficiency, clustering coefficient, transitivity, modularity, assortativity, and small‐worldness index from the connectivity matrix using a graph theory (Mijalkov et al., 2017). To compare the differences between the groups in the intrinsic local amygdala‐hippocampal network, we used the measure of the betweenness centrality, which defined the nodes as the regions of interest in the 18 nuclei in the amygdala and 38 hippocampal subfields. We investigated the differences in these global and local network measures between patients with JME and healthy controls. Then, to confirm our findings, we also performed graph theoretical analysis using binary undirected graphs at a fixed density of connections (density ranging from 10% to 90% in increments of 10%) when there were statistical significances with a weighted connectivity analysis.

### 2.4 Statistical analysis

The factors were compared and analyzed using the Chi‐squared test for categorical variables, and the Student's *t*‐test for continuous variables. The global and local amygdala‐hippocampal network measures were compared by testing the statistical significance of the differences using nonparametric permutation tests with 1000 permutations, because we could obtain the measures as a group level, not individual subjects. A two‐side *p*‐value of less than .05 indicated statistical significance. Multiple corrections with a false discovery rate were performed in the analysis of individual volumes of the nuclei in the amygdala, the hippocampal subfields, and the intrinsic local amygdala‐hippocampal network. In addition, we analyzed the correlation analysis between the volumes of nuclei in the amygdala and the hippocampal subfields, and the clinical factors including age at the time of MRI, age of seizure onset, and duration of epilepsy using a Pearson correlation test. All statistical tests were performed using MedCalc® (MedCalc Software version 19.4.0, Ostend, Belgium; https://www.medcalc.org; 2020).

## 3 RESULTS

### 3.1 Subjects’ demographic and clinical characteristics

Table 1 lists the demographic and clinical characteristics of the subjects. The age and sex ratios were not much different between patients with JME and healthy subjects (*p *= .480 and *p *= .712, respectively). In patients with JME, the mean age of seizure onset was 17.5 ± 4.6 years, and the median duration between the first seizure and the time of brain MRI was 38 (0–372) months.

**TABLE 1:** Subjects’ demographic and clinical characteristics

Variables	Patients with JME (*n* = 35)	Healthy controls (*n* = 34)	*p*‐value
Mean age, years (±SD)	23.8 ± 6.0	24.7 ± 3.0	.480
Male, *n* (%)	18 (51.4)	19 (55.8)	.712
Mean age of seizure onset, years (±SD)	17.5 ± 4.6		
Median duration between the first seizure and the time of brain MRI taken, months (range)	38 (0–372)		
Myoclonic seizures, *n* (%)	35 (100.0)		
Absence seizures, *n* (%)	9 (25.7)		
Generalized tonic–clonic seizures, *n* (%)	30 (85.7)		

JME, juvenile myoclonic epilepsy; SD, standard deviation.

### 3.2 Volumes of nuclei in the amygdala and the hippocampal subfields

The total volumes of the amygdala and the hippocampus of patients with JME were not very different from those in the healthy controls (right amygdala, 0.117% vs. 0.124%, *p *= .069; left amygdala, 0.116% vs. 0.119%, *p *= .326; right hippocampus, 0.230% vs. 0.241%, *p *= .104; left hippocampus, 0.230% vs. 0.239%, *p *= .248, respectively) (Supporting Information 1). Furthermore, there were no significant volume differences in the nuclei of the amygdala and the hippocampal subfields between patients with JME and healthy controls (Supporting Information 1).

### 3.3 The intrinsic amygdala‐hippocampal global network

**FIGURE 2:**
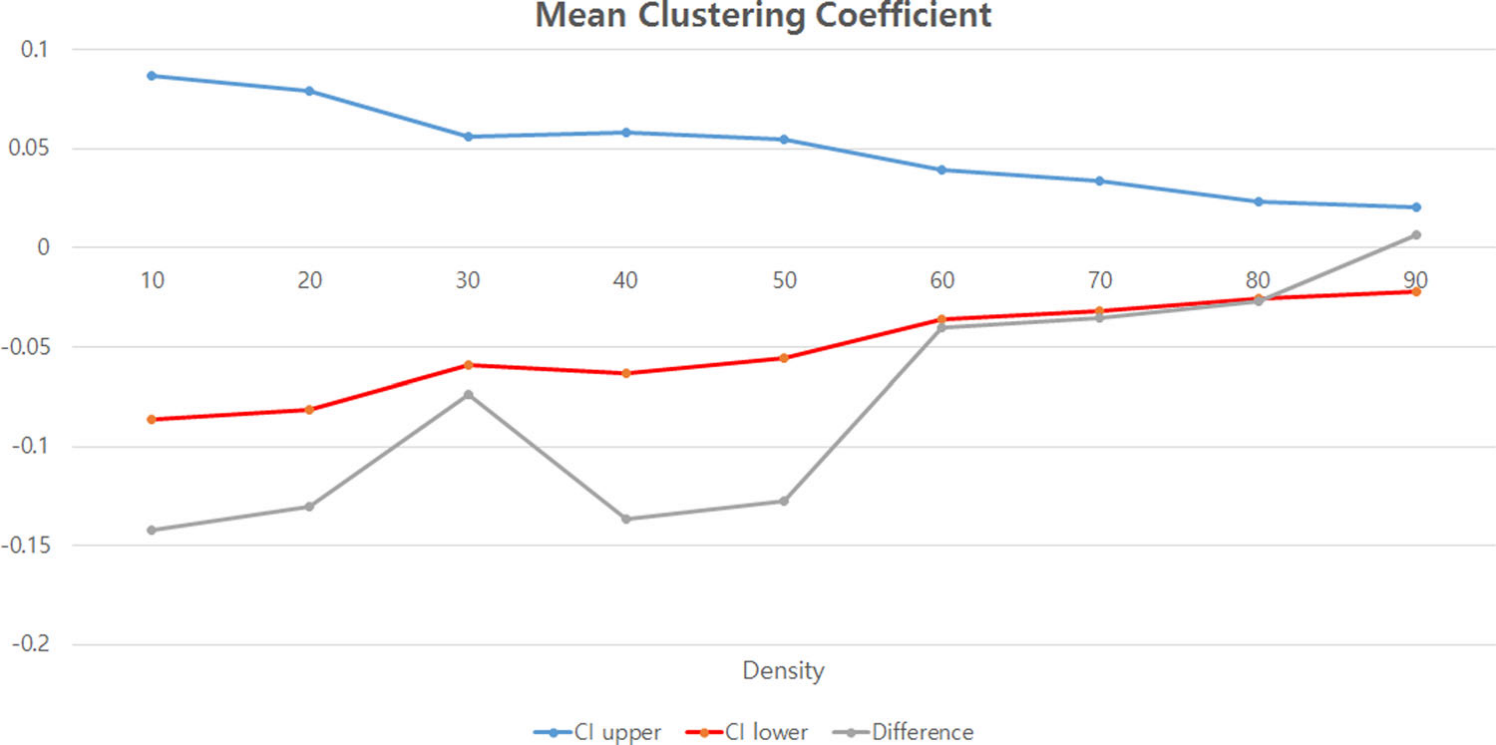
Differences in the network measures of mean clustering coefficient between patients with juvenile myoclonic epilepsy and healthy controls. The blue and red lines indicate the upper and lower limits of the 95% confidence interval of the differences between patients with juvenile myoclonic epilepsy and healthy controls, and the gray lines show the mean difference values between the groups according to fixed density

Table 2 presents the intrinsic amygdala‐hippocampal global network. There were significant differences in the global network between patients with JME and healthy controls. The mean clustering coefficient was significantly decreased in patients with JME compared to healthy controls (0.473 vs. 0.653, *p *= .047). Graph theoretical analysis using binary undirected graphs at a fixed density range of connections also showed significant differences in the mean clustering coefficient between them (Figure 2).

**TABLE 2:** Differences in intrinsic amygdala‐hippocampal global network between patients with juvenile myoclonic epilepsy and healthy controls

Measures	Patients with JME	Healthy controls	Differences	CI lower	CI upper	*p*‐value
Average degree	57.633	58.933	1.300	−2.033	1.937	.129
Average strength	29.157	39.389	10.232	−11.771	11.576	.075
Radius	3.254	2.665	−0.590	−1.014	1.071	.164
Diameter	5.494	4.340	−1.154	−1.741	1.763	.135
Eccentricity	4.406	3.167	−1.239	−1.348	1.324	.063
Characteristic path length	2.235	1.613	−0.622	−0.732	0.712	.064
Global efficiency	0.517	0.674	0.157	−0.182	0.165	.056
Local efficiency	2.117	3.545	1.428	−1.573	1.552	.066
Mean clustering coefficient	0.474	0.653	0.180	−0.216	0.178	.047
Transitivity	0.717	0.980	0.263	−0.315	0.306	.069
Modularity	0.039	0.025	−0.014	−0.033	0.036	.221
Assortativity	−0.019	−0.022	−0.003	−0.043	0.040	.443
Small‐worldness index	0.935	0.978	0.043	−0.059	0.056	.107

JME, juvenile myoclonic epilepsy; CI, 95% confidence interval of the differences between the groups.

### 3.4 The intrinsic amygdala‐hippocampal local network

**FIGURE 3:**
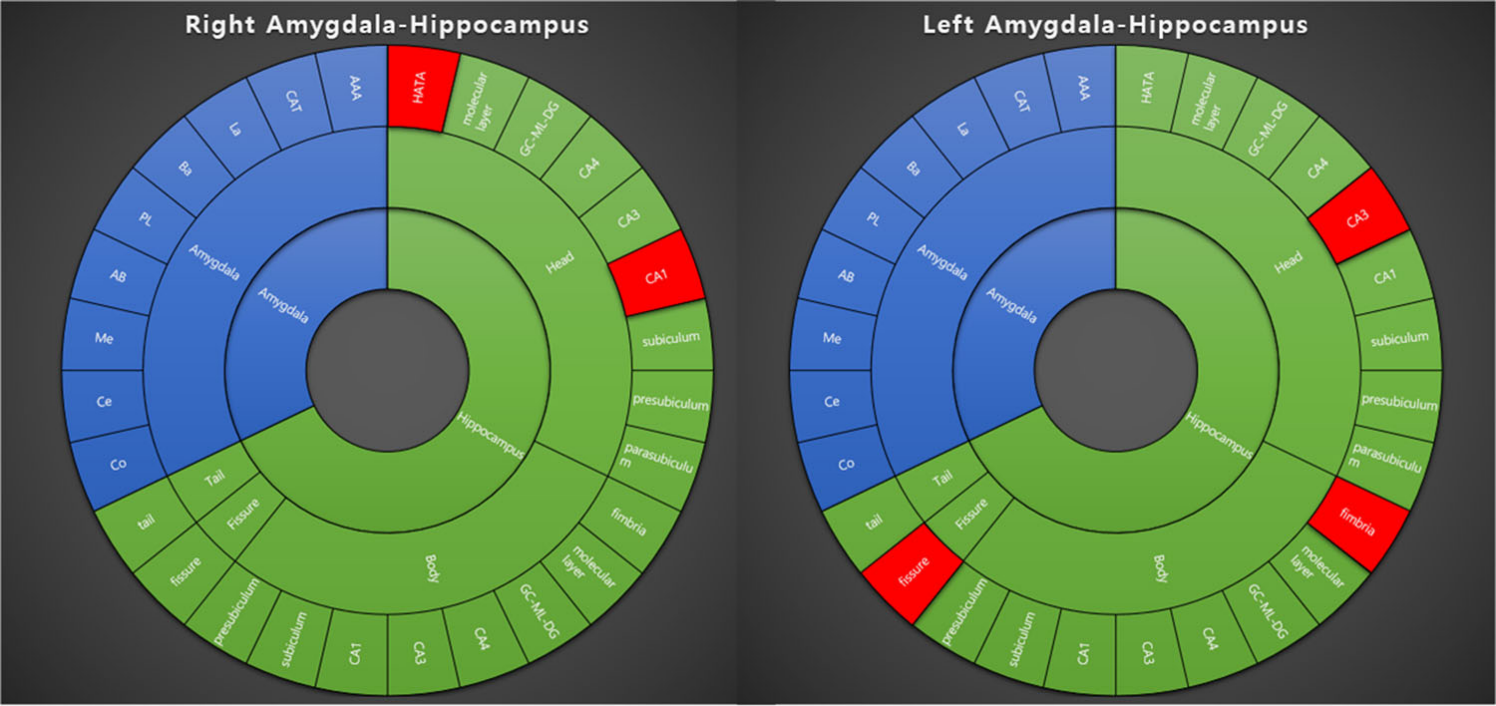
The differences in the intrinsic amygdala‐hippocampal local network between patients with juvenile myoclonic epilepsy and healthy controls. Red‐colored areas reveal nodes with significantly different local networks. AAA, anterior amygdala area; CAT, cortico‐amygdaloid transition area; La, lateral nucleus; Ba, basal nucleus; PL, paralaminar nucleus; AB, accessory basal nucleus; Me, medial nucleus; Ce, central nucleus; Co, cortical nucleus

Figure 3 presents the differences in the intrinsic amygdala‐hippocampal local network between patients with JME and healthy controls (Supporting Information 2). Some regions in the hippocampal subfields showed significant differences in local networks between the two groups. The betweenness centrality of the right CA1‐head, right hippocampus–amygdala‐transition area, left hippocampal fissure, left fimbria, and left CA3‐head were increased in patients with JME compared to healthy controls (0.0076 vs. 0.0000, *p *= .011; 0.0005 vs. 0.0000, *p *= .041; 0.0053 vs. 0.0000, *p *= .020; 0.0012 vs. 0.0000, *p *= .024; 0.0005 vs. 0.0000, *p *= .027, respectively).

### 3.5 Correlation analysis between the clinical factors and volumes of nuclei in the amygdala and the hippocampal subfields

Age was significantly correlated with the volumes of the right CA3 body and right central nucleus (*r* = −0.362, *p *= .033; *r* = −0.446, *p *= .007, respectively). The age of seizure onset was significantly correlated with the volumes of the left medial nucleus, right paralaminar nucleus, and right subiculum head (*r* = 0.369, *p *= .032; *r* = 0.397, *p *= .020; *r* = 0.352, *p *= .041, respectively). The duration of epilepsy was significantly correlated with the volumes of the right anterior amygdaloid area and right central nucleus (*r* = −0.406, *p *= .016; *r* = −0.432, *p *= .010, respectively) (Supporting Information 3).

## 4 DISCUSSION

The main finding of this study was that the intrinsic amygdala‐hippocampal network differed in patients with JME compared to healthy controls, although the volumes of the individual nuclei of the amygdala and the hippocampal subfields were not different between the two groups.

Graph theory is a useful tool for deciphering structural and functional networks in the brain (Masuda et al., 2018). Of the measures in the graph theory, the clustering coefficient can be used to quantify the abundance of connected triangles in a network and is therefore a major descriptive network statistic (Masuda et al., 2018). It is used to measure local connectivity and segregation, which is associated with efficient recurrent processing in closed feedback loops and efficient information exchanges (Haneef & Chiang, 2014). In the intrinsic amygdala‐hippocampal global network, we found that the mean clustering coefficient was significantly decreased in patients with JME. These findings suggested that the local efficiency of the amygdala‐hippocampal network was decreased, revealing the dysfunction of amygdala‐hippocampal structures in patients with JME (Haneef & Chiang, 2014). In addition, we found that, in the intrinsic amygdala‐hippocampal local network, the betweenness centrality of several regions in the hippocampus was increased in patients with JME compared to healthy controls. The betweenness centrality is a measure used to identify nodes located on the most traveled paths, based on the number of shortest paths that pass through a node (Haneef & Chiang, 2014). This finding suggests the increased local connectivity among the nodes of hippocampus. Taken all together, in the present study, we found that the alterations in the intrinsic amygdala‐hippocampal network were associated with increased local connectivity and diminished global connectivity.

Several lines of evidence suggest that the amygdala–hippocampus may be involved in the epileptogenic network of JME. A recent study with multi‐modal MRI and neuropsychological data investigated hippocampal structure and function in patients with JME (Caciagli et al., 2019). The study revealed a reduction in left hippocampal volume, with increased frequency of hippocampal mal‐rotation compared to healthy controls. Functional mapping also revealed atypical patterns of hippocampal activation, pointing to abnormal recruitment during verbal encoding (Caciagli et al., 2019). Another study with diffusion tensor imaging analyzed the microstructural alterations of subcortical gray matter in patients with JME, and it found that the patients with JME had a significant increase in mean diffusivity of the bilateral hippocampus (Kim et al., 2018). Increased mean diffusivity indicates enlarged extracellular space due to altered cytoarchitecture, resulting from the disruption of white matter tracts connecting the hippocampus and other structures (Kim et al., 2018). Another study investigated the metabolic differences in hippocampus between patients with JME and healthy controls using magnetic resonance spectroscopy (Ristic et al., 2011). The study revealed decreased levels of *N*‐acetyl‐aspartate throughout the left hippocampus (Ristic et al., 2011). Furthermore, the study of structural and functional connectivity showed altered nodal topological characteristics of amygdala in patients with genetic generalized epilepsy (Zhang et al., 2011). The authors stated that regions playing an important role in the pathogenesis of epilepsy may display abnormal hub properties in network analysis (Zhang et al., 2011). Another study performed mice haploinsufficient in *Brd2*, a gene associated with JME, and found decreased numbers of GABA neurons in the basolateral amygdala, which was consistent with the increase in aggressive behavior (Chachua et al., 2014). In addition, a previous study which investigated the alterations in the subcortical structures of patients with genetic generalized epilepsy, indicated the relationship between presence of focal atrophy in the basolateral amygdala and drug resistance (Li et al., 2021). Taken together, all of these findings suggested that the amygdala–hippocampus may play a key role in the pathogenesis of JME.

Several studies reported cognitive impairment in patients with JME. A study in Korea revealed that verbal learning, attention, and verbal fluency were significantly lower in patients with JME than in healthy controls, although no differences in general intellectual ability and mood were detected (Kim et al., 2007). Another study also showed cognitive impairments in patients with JME, including full‐scale IQ, processing speed, visual memory, verbal fluency and inhibition, and executive function (Thomas et al., 2014). Sezikli et al. analyzed the relationship between cognitive function and interictal epileptiform discharges in patients with JME, and reported that patients with JME scored worse than the control group in attention, memory, and frontal lobe functions. In addition, they demonstrated that the patients with asymmetrical generalized discharges had lower scores of cognitive function than those with symmetrical generalized discharges (Sezikli et al., 2018). Furthermore, previous researches have shown that amygdala plays an important role in memory (Bass et al., 2012; Inman et al., 2018; Zhang & Li, 2018). A study of patients with epilepsy found that amygdala stimulation via intracranial depth electrodes led to reliably improved memory with endogenous memory prioritization processes, in the absence of emotional input (Inman et al., 2018). Another study with rats indicated that the basolateral complex of the amygdala stimulation could enhance memory for individual events, a necessary ability for the amygdala to modulate episodic memory effectively (Bass et al., 2012). All of these previous studies suggested cognitive dysfunction, especially memory impairment, in patients with JME, which could be attributed to changes in the intrinsic amygdala‐hippocampal network. A previous structure–function analysis revealed a linear relationship between quantitative measures of left hippocampal positioning and activation for successful verbal encoding. The findings provide direct evidence suggesting that morphological features of the hippocampus may modulate its functional recruitment during a memory task (Caciagli et al., 2019).

This was the first study to investigate the volume differences in the nuclei of the amygdala and the hippocampal subfields and alterations of the intrinsic amygdala‐hippocampal network in patients with JME compared to healthy controls. We successfully demonstrated the changes in the intrinsic amygdala‐hippocampal global and local network in patients with JME. In addition, we only enrolled patients with newly diagnosed JME to exclude the effects of the antiseizure medications on the structural volumes and brain network (Pang et al., 2020).

However, the study has several limitations. First, this study was retrospectively conducted in a single center. Further multi‐center studies involving large sample sizes may be needed to corroborate our findings. Second, we could not obtain the cognitive function data using neuropsychological test in patients with JME. Thus, the correlation analysis between the volumes or network measures and cognitive functions in patients with JME could not be performed, and the relationship between the network changes and memory dysfunctions in patients with JME could not be elucidated. Third, it was difficult to determine whether these network changes originated as a result of the results of recurrent seizures or contributed to JME, because this study was a cross‐sectional one. Fourth, we could not obtain the data for the handedness scale and education level in patients with JME, which might affect brain connectivity. Last, we only investigated the intrinsic amygdala‐hippocampal network using a co‐variance analysis based on structural volumes. Thus, further studies using diffusion tensor imaging, functional MRI, EEG, or MEG are needed to provide further direct evidence of alterations in the structural or functional connectivity in patients with JME.

## 5 CONCLUSION

The intrinsic amygdala‐hippocampal global and local network differed in patients with JME, compared to healthy controls, which may be related to the pathogenesis of JME and memory dysfunction in patients with JME.

## CONFLICT OF INTEREST

All the authors have no conflict of interest to declare.

## AUTHOR CONTRIBUTIONS

*Conception and design*: Dong Ah Lee, Junghae Ko, and Kang Min Park. *Acquisition of data, analysis, and interpretation of data*: Ho‐Joon Lee, Hyung Chan Kim, Bong Soo Park, Sihyung Park, Il Hwan Kim, Jin Han Park, Yoo Jin Lee, and Kang Min Park. *Drafting the manuscript or revising*: Dong Ah Lee, Junghae Ko, and Kang Min Park. *Final approval*: Kang Min Park.

### PEER REVIEW

The peer review history for this article is available at https://publons.com/publon/10.1002/brb3.2274.

## Supporting information

Table S1

Table S2

Table S3

## Data Availability

The data that support the findings of this study are available from the corresponding author upon reasonable request.
