# Supplementary material for: Alterations of the intrinsic amygdala‐hippocampal network in juvenile myoclonic epilepsy
Source: Brain Behav. 2021 Jul 5;11(8):e2274. doi: 10.1002/brb3.2274 (PMC8413739; doi:10.1002/brb3.2274)
Supplement: Supplementary file 1 — Table S1 [file BRB3-11-e2274-s001.docx]

**Supplementary 1**. Volumes of the nuclei of the amygdala and the hippocampal subfields

| Regions | Patients with JME | | Healthy Controls | |  |
| --- | --- | --- | --- | --- | --- |
|  | Mean | SD | Mean | SD | *p*-value |
| Amgydala |  |  |  |  |  |
| Accessory_Basal_nucleus_Lt | 0.0179 | 0.0019 | 0.0189 | 0.0025 | 0.0635 |
| Accessory_Basal_nucleus_Rt | 0.0181 | 0.0020 | 0.0193 | 0.0029 | 0.0396 |
| Anterior_amygdaloid_area_Lt | 0.0039 | 0.0005 | 0.0040 | 0.0006 | 0.6605 |
| Anterior_amygdaloid_area_Rt | 0.0041 | 0.0005 | 0.0044 | 0.0006 | 0.0661 |
| Basal_nucleus_Lt | 0.0296 | 0.0031 | 0.0303 | 0.0039 | 0.4022 |
| Basal_nucleus_Rt | 0.0300 | 0.0034 | 0.0316 | 0.0046 | 0.1017 |
| Central_nucleus_Lt | 0.0028 | 0.0004 | 0.0029 | 0.0005 | 0.5531 |
| Central_nucleus_Rt | 0.0032 | 0.0006 | 0.0034 | 0.0006 | 0.1464 |
| Cortical_nucleus_Lt | 0.0019 | 0.0002 | 0.0019 | 0.0003 | 0.4742 |
| Cortical_nucleus_Rt | 0.0019 | 0.0003 | 0.0019 | 0.0003 | 0.8665 |
| Corticoamygdaloid_transition_area_Lt | 0.0122 | 0.0014 | 0.0128 | 0.0019 | 0.1050 |
| Corticoamygdaloid_transition_area_Rt | 0.0116 | 0.0015 | 0.0125 | 0.0020 | 0.0548 |
| Lateral_nucleus_Lt | 0.0434 | 0.0043 | 0.0441 | 0.0061 | 0.5668 |
| Lateral_nucleus_Rt | 0.0443 | 0.0054 | 0.0469 | 0.0065 | 0.0731 |
| Medial_nucleus_Lt | 0.0015 | 0.0004 | 0.0013 | 0.0002 | 0.0414 |
| Medial_nucleus_Rt | 0.0016 | 0.0004 | 0.0014 | 0.0003 | 0.2015 |
| Paralaminar_nucleus_Lt | 0.0033 | 0.0004 | 0.0034 | 0.0004 | 0.4354 |
| Paralaminar_nucleus_Rt | 0.0032 | 0.0004 | 0.0033 | 0.0005 | 0.3739 |
| Hippocampus |  |  |  |  |  |
| CA1_body_Lt | 0.0077 | 0.0014 | 0.0080 | 0.0014 | 0.2744 |
| CA1_body_Rt | 0.0081 | 0.0011 | 0.0090 | 0.0017 | 0.0084 |
| CA1_head_Lt | 0.0342 | 0.0046 | 0.0359 | 0.0048 | 0.1422 |
| CA1_head_Rt | 0.0353 | 0.0044 | 0.0376 | 0.0061 | 0.0822 |
| CA3_body_Lt | 0.0056 | 0.0009 | 0.0057 | 0.0010 | 0.5724 |
| CA3_body_Rt | 0.0061 | 0.0009 | 0.0064 | 0.0011 | 0.2024 |
| CA3_head_Lt | 0.0083 | 0.0016 | 0.0086 | 0.0016 | 0.3724 |
| CA3_head_Rt | 0.0085 | 0.0011 | 0.0090 | 0.0014 | 0.1202 |
| CA4_body_Lt | 0.0080 | 0.0012 | 0.0082 | 0.0015 | 0.5762 |
| CA4_body_Rt | 0.0080 | 0.0008 | 0.0084 | 0.0013 | 0.0998 |
| CA4_head_Lt | 0.0087 | 0.0015 | 0.0089 | 0.0014 | 0.4906 |
| CA4_head_Rt | 0.0089 | 0.0010 | 0.0094 | 0.0015 | 0.1054 |
| fimbria_Lt | 0.0060 | 0.0010 | 0.0061 | 0.0012 | 0.7398 |
| fimbria_Rt | 0.0053 | 0.0009 | 0.0060 | 0.0013 | 0.0137 |
| granule cell layer of dentate gyrus _body_Lt | 0.0091 | 0.0014 | 0.0093 | 0.0016 | 0.5351 |
| granule cell layer of dentate gyrus _body_Rt | 0.0089 | 0.0009 | 0.0094 | 0.0015 | 0.0789 |
| granule cell layer of dentate gyrus _head_Lt | 0.0105 | 0.0017 | 0.0109 | 0.0017 | 0.4037 |
| granule cell layer of dentate gyrus _head_Rt | 0.0108 | 0.0013 | 0.0114 | 0.0018 | 0.1182 |
| hippocampus-amygdala-transition _Lt | 0.0039 | 0.0007 | 0.0044 | 0.0009 | 0.0085 |
| hippocampus-amygdala-transition _Rt | 0.0037 | 0.0008 | 0.0043 | 0.0008 | 0.0027 |
| hippocampal_fissure_Lt | 0.0083 | 0.0015 | 0.0084 | 0.0015 | 0.7503 |
| hippocampal_fissure_Rt | 0.0086 | 0.0013 | 0.0089 | 0.0019 | 0.3258 |
| Hippocampal_tail_Lt | 0.0357 | 0.0039 | 0.0375 | 0.0065 | 0.1758 |
| Hippocampal_tail_Rt | 0.0357 | 0.0036 | 0.0361 | 0.0066 | 0.7646 |
| molecular_layer_HP_body_Lt | 0.0149 | 0.0021 | 0.0155 | 0.0026 | 0.3543 |
| molecular_layer_HP_body_Rt | 0.0150 | 0.0014 | 0.0158 | 0.0025 | 0.0810 |
| molecular_layer_HP_head_Lt | 0.0222 | 0.0028 | 0.0232 | 0.0032 | 0.1505 |
| molecular_layer_HP_head_Rt | 0.0228 | 0.0027 | 0.0242 | 0.0037 | 0.0772 |
| parasubiculum_Lt | 0.0043 | 0.0009 | 0.0043 | 0.0009 | 0.8386 |
| parasubiculum_Rt | 0.0041 | 0.0007 | 0.0041 | 0.0009 | 0.9848 |
| presubiculum_body_Lt | 0.0116 | 0.0019 | 0.0119 | 0.0025 | 0.5799 |
| presubiculum_body_Rt | 0.0104 | 0.0013 | 0.0102 | 0.0021 | 0.6497 |
| presubiculum_head_Lt | 0.0097 | 0.0012 | 0.0098 | 0.0014 | 0.7403 |
| presubiculum_head_Rt | 0.0096 | 0.0012 | 0.0098 | 0.0017 | 0.4378 |
| subiculum_body_Lt | 0.0170 | 0.0026 | 0.0173 | 0.0036 | 0.6739 |
| subiculum_body_Rt | 0.0159 | 0.0016 | 0.0165 | 0.0030 | 0.3314 |
| subiculum_head_Lt | 0.0129 | 0.0019 | 0.0135 | 0.0021 | 0.2244 |
| subiculum_head_Rt | 0.0132 | 0.0019 | 0.0143 | 0.0024 | 0.0524 |
| Whole_hippocampal_body_Lt | 0.0799 | 0.0108 | 0.0821 | 0.0139 | 0.4762 |
| Whole_hippocampal_body_Rt | 0.0776 | 0.0064 | 0.0817 | 0.0129 | 0.0949 |
| Whole_hippocampal_head_Lt | 0.1148 | 0.0144 | 0.1196 | 0.0162 | 0.1961 |
| Whole_hippocampal_head_Rt | 0.1169 | 0.0135 | 0.1241 | 0.0190 | 0.0753 |
