# Supplementary material for: Alterations of the intrinsic amygdala‐hippocampal network in juvenile myoclonic epilepsy
Source: Brain Behav. 2021 Jul 5;11(8):e2274. doi: 10.1002/brb3.2274 (PMC8413739; doi:10.1002/brb3.2274)
Supplement: Supplementary file 2 — Table S2 [file BRB3-11-e2274-s002.docx]

**Supplementary 2.** The intrinsic amygdala-hippocampal local network

|  | Patients with JME | Healthy controls | Differences | CI upper | CI lower | *p*-value |
| --- | --- | --- | --- | --- | --- | --- |
| Amygdala |  |  |  |  |  |  |
| Lateral_nucleus_Lt | 0.0023 | 0.0000 | -0.0023 | -0.0105 | 0.0093 | 0.617 |
| Basal_nucleus_Lt | 0.0082 | 0.0012 | -0.0070 | -0.0141 | 0.0155 | 0.335 |
| Accessory_Basal_nucleus_Lt | 0.0193 | 0.0082 | -0.0111 | -0.0257 | 0.0287 | 0.394 |
| Anterior_amygdaloid_area_Lt | 0.0000 | 0.0000 | 0.0000 | -0.0023 | 0.0023 | 0.505 |
| Central_nucleus_Lt | 0.0018 | 0.0023 | 0.0006 | -0.0082 | 0.0093 | 0.914 |
| Medial_nucleus_Lt | 0.0000 | 0.0000 | 0.0000 | -0.0006 | 0.0006 | 0.153 |
| Cortical_nucleus_Lt | 0.0269 | 0.0181 | -0.0088 | -0.0228 | 0.0211 | 0.433 |
| Corticoamygdaloid_transition area_Lt | 0.0000 | 0.0000 | 0.0000 | -0.0017 | 0.0017 | 0.575 |
| Paralaminar_nucleus_Lt | 0.0012 | 0.0058 | 0.0047 | -0.0064 | 0.0082 | 0.247 |
| Lateral_nucleus_Rt | 0.0012 | 0.0000 | -0.0012 | -0.0062 | 0.0079 | 0.492 |
| Basal_nucleus_Rt | 0.0047 | 0.0035 | -0.0012 | -0.0088 | 0.0091 | 0.762 |
| Accessory_Basal_nucleus_Rt | 0.0432 | 0.0070 | -0.0362 | -0.0336 | 0.0322 | 0.070 |
| Anterior_amygdaloid_area_Rt | 0.0000 | 0.0000 | 0.0000 | -0.0006 | 0.0006 | 0.147 |
| Central_nucleus_Rt | 0.0000 | 0.0006 | 0.0006 | -0.0108 | 0.0123 | 0.790 |
| Medial_nucleus_Rt | 0.0000 | 0.0000 | 0.0000 | -0.0018 | 0.0012 | 0.503 |
| Cortical_nucleus_Rt | 0.0058 | 0.0000 | -0.0058 | -0.0275 | 0.0281 | 0.575 |
| Corticoamygdaloid_transition area_Rt | 0.0000 | 0.0000 | 0.0000 | -0.0035 | 0.0041 | 0.801 |
| Paralaminar_nucleus_Rt | 0.0058 | 0.0012 | -0.0047 | -0.0102 | 0.0111 | 0.425 |
| Hippocampus |  |  |  |  |  |  |
| Hippocampal_tail_Lt | 0.0006 | 0.0000 | -0.0006 | -0.0053 | 0.0058 | 0.404 |
| subiculum_body_Lt | 0.0018 | 0.0000 | -0.0018 | -0.0053 | 0.0058 | 0.354 |
| CA1_body_Lt | 0.0000 | 0.0000 | 0.0000 | -0.0023 | 0.0029 | 0.412 |
| subiculum_head_Lt | 0.0035 | 0.0000 | -0.0035 | -0.0041 | 0.0032 | 0.106 |
| hippocampal_fissure_Lt | 0.0053 | 0.0000 | -0.0053 | -0.0018 | 0.0018 | 0.020 |
| presubiculum_head_Lt | 0.0029 | 0.0000 | -0.0029 | -0.0126 | 0.0123 | 0.484 |
| CA1_head_Lt | 0.0006 | 0.0023 | 0.0018 | -0.0085 | 0.0088 | 0.650 |
| presubiculum_body_Lt | 0.0000 | 0.0000 | 0.0000 | -0.0023 | 0.0017 | 0.431 |
| parasubiculum_Lt | 0.0000 | 0.0006 | 0.0006 | -0.0026 | 0.0032 | 0.454 |
| molecular_layer_HP_head_Lt | 0.0076 | 0.0041 | -0.0035 | -0.0155 | 0.0164 | 0.666 |
| molecular_layer_HP_body_Lt | 0.0129 | 0.0000 | -0.0129 | -0.0108 | 0.0105 | 0.071 |
| granule cell layer of dentate gyrus_head_Lt | 0.0000 | 0.0000 | 0.0000 | -0.0023 | 0.0023 | 0.519 |
| CA3_body_Lt | 0.0006 | 0.0000 | -0.0006 | -0.0026 | 0.0041 | 0.220 |
| granule cell layer of dentate gyrus_body_Lt | 0.0006 | 0.0000 | -0.0006 | -0.0018 | 0.0024 | 0.288 |
| CA4_head_Lt | 0.0023 | 0.0000 | -0.0023 | -0.0018 | 0.0018 | 0.065 |
| CA4_body_Lt | 0.0041 | 0.0000 | -0.0041 | -0.0029 | 0.0029 | 0.059 |
| fimbria_Lt | 0.0012 | 0.0000 | -0.0012 | -0.0006 | 0.0006 | 0.024 |
| CA3_head_Lt | 0.0006 | 0.0000 | -0.0006 | 0.0000 | 0.0000 | 0.027 |
| hippocampus-amygdala-transition_Lt | 0.0006 | 0.0000 | -0.0006 | -0.0012 | 0.0012 | 0.231 |
| Whole_hippocampal_body_Lt | 0.0094 | 0.0012 | -0.0082 | -0.0135 | 0.0161 | 0.256 |
| Whole_hippocampal_head_Lt | 0.0175 | 0.0006 | -0.0169 | -0.0117 | 0.0126 | 0.038 |
| Hippocampal_tail_Rt | 0.0006 | 0.0000 | -0.0006 | -0.0012 | 0.0015 | 0.123 |
| subiculum_body_Rt | 0.0006 | 0.0006 | 0.0000 | -0.0064 | 0.0070 | 0.783 |
| CA1_body_Rt | 0.0000 | 0.0000 | 0.0000 | -0.0006 | 0.0006 | 0.137 |
| subiculum_head_Rt | 0.0012 | 0.0006 | -0.0006 | -0.0053 | 0.0067 | 0.715 |
| hippocampal_fissure_Rt | 0.0000 | 0.0006 | 0.0006 | -0.0035 | 0.0041 | 0.509 |
| presubiculum_head_Rt | 0.0111 | 0.0012 | -0.0099 | -0.0234 | 0.0213 | 0.507 |
| CA1_head_Rt | 0.0076 | 0.0000 | -0.0076 | -0.0029 | 0.0029 | 0.011 |
| presubiculum_body_Rt | 0.0000 | 0.0000 | 0.0000 | -0.0012 | 0.0006 | 0.195 |
| parasubiculum_Rt | 0.0000 | 0.0000 | 0.0000 | 0.0000 | 0.0000 | 0.089 |
| molecular_layer_HP_head_Rt | 0.0023 | 0.0000 | -0.0023 | -0.0058 | 0.0058 | 0.383 |
| molecular_layer_HP_body_Rt | 0.0029 | 0.0000 | -0.0029 | -0.0140 | 0.0146 | 0.516 |
| granule cell layer of dentate gyrus_head_Rt | 0.0000 | 0.0000 | 0.0000 | -0.0006 | 0.0012 | 0.326 |
| CA3_body_Rt | 0.0006 | 0.0000 | -0.0006 | -0.0006 | 0.0006 | 0.066 |
| granule cell layer of dentate gyrus_body_Rt | 0.0000 | 0.0006 | 0.0006 | -0.0029 | 0.0029 | 0.620 |
| CA4_head_Rt | 0.0000 | 0.0000 | 0.0000 | -0.0012 | 0.0017 | 0.436 |
| CA4_body_Rt | 0.0018 | 0.0000 | -0.0018 | -0.0023 | 0.0029 | 0.133 |
| fimbria_Rt | 0.0000 | 0.0000 | 0.0000 | -0.0006 | 0.0006 | 0.109 |
| CA3_head_Rt | 0.0000 | 0.0000 | 0.0000 | 0.0000 | 0.0000 | 0.054 |
| hippocampus-amygdala-transition_Rt | 0.0006 | 0.0000 | -0.0006 | 0.0000 | 0.0006 | 0.041 |
| Whole_hippocampal_body_Rt | 0.0292 | 0.0018 | -0.0275 | -0.0169 | 0.0166 | 0.012 |
| Whole_hippocampal_head_Rt | 0.0029 | 0.0029 | 0.0000 | -0.0070 | 0.0076 | 0.937 |

CI: 95% confidence interval of the differences between the groups
