# Supplementary material for: Alterations of the intrinsic amygdala‐hippocampal network in juvenile myoclonic epilepsy
Source: Brain Behav. 2021 Jul 5;11(8):e2274. doi: 10.1002/brb3.2274 (PMC8413739; doi:10.1002/brb3.2274)
Supplement: Supplementary file 3 — Table S3 [file BRB3-11-e2274-s003.docx]

**Supplementary 3**. Correlation analysis between the clinical factors and volumes of nuclei in the amygdala and the hippocampal subfields

|  | Age | Age of seizure onset | Duration of epilepsy |
| --- | --- | --- | --- |
| Accessory_Basal_nucleus_Lt | 0.127 | 0.338 | -0.082 |
|  | 0.467 | 0.051 | 0.641 |
| Accessory_Basal_nucleus_Rt | -0.112 | 0.288 | -0.282 |
|  | 0.522 | 0.099 | 0.101 |
| Anterior_amygdaloid_area_Lt | 0.167 | 0.163 | 0.000 |
|  | 0.337 | 0.357 | 0.999 |
| Anterior_amygdaloid_area_Rt | -0.260 | 0.217 | -0.406 |
|  | 0.131 | 0.217 | 0.016 |
| Basal_nucleus_Lt | 0.003 | 0.286 | -0.146 |
|  | 0.988 | 0.101 | 0.403 |
| Basal_nucleus_Rt | -0.152 | 0.335 | -0.322 |
|  | 0.383 | 0.053 | 0.059 |
| CA1_body_Lt | 0.031 | 0.076 | -0.039 |
|  | 0.858 | 0.667 | 0.825 |
| CA1_body_Rt | -0.139 | 0.057 | -0.239 |
|  | 0.427 | 0.750 | 0.167 |
| CA1_head_Lt | 0.030 | 0.195 | -0.138 |
|  | 0.866 | 0.269 | 0.429 |
| CA1_head_Rt | -0.113 | 0.192 | -0.247 |
|  | 0.520 | 0.276 | 0.153 |
| CA3_body_Lt | 0.086 | 0.138 | -0.009 |
|  | 0.624 | 0.436 | 0.957 |
| CA3_body_Rt | -0.362 | -0.096 | -0.284 |
|  | 0.033 | 0.588 | 0.098 |
| CA3_head_Lt | -0.123 | 0.042 | -0.126 |
|  | 0.481 | 0.811 | 0.472 |
| CA3_head_Rt | -0.182 | 0.010 | -0.134 |
|  | 0.295 | 0.954 | 0.444 |
| CA4_body_Lt | -0.106 | -0.018 | -0.096 |
|  | 0.546 | 0.919 | 0.583 |
| CA4_body_Rt | -0.215 | -0.077 | -0.148 |
|  | 0.216 | 0.666 | 0.397 |
| CA4_head_Lt | -0.150 | 0.039 | -0.164 |
|  | 0.391 | 0.827 | 0.346 |
| CA4_head_Rt | -0.144 | 0.024 | -0.136 |
|  | 0.411 | 0.894 | 0.438 |
| Central_nucleus_Lt | -0.162 | 0.105 | -0.235 |
|  | 0.352 | 0.555 | 0.174 |
| Central_nucleus_Rt | -0.446 | 0.089 | -0.432 |
|  | 0.007 | 0.617 | 0.010 |
| Cortical_nucleus_Lt | 0.149 | 0.332 | -0.141 |
|  | 0.394 | 0.055 | 0.419 |
| Cortical_nucleus_Rt | 0.040 | 0.241 | -0.187 |
|  | 0.819 | 0.171 | 0.282 |
| Corticoamygdaloid_transition_area_Lt | 0.146 | 0.351 | -0.035 |
|  | 0.402 | 0.042 | 0.841 |
| Corticoamygdaloid_transition_area_Rt | -0.019 | 0.326 | -0.202 |
|  | 0.915 | 0.060 | 0.246 |
| fimbria_Lt | 0.072 | -0.063 | 0.143 |
|  | 0.679 | 0.724 | 0.411 |
| fimbria_Rt | 0.207 | 0.271 | 0.085 |
|  | 0.233 | 0.122 | 0.627 |
| granule cell layer of dentate gyrus _body_Lt | -0.082 | -0.055 | -0.067 |
|  | 0.638 | 0.756 | 0.702 |
| granule cell layer of dentate gyrus _body_Rt | -0.165 | -0.046 | -0.133 |
|  | 0.343 | 0.795 | 0.445 |
| granule cell layer of dentate gyrus _head_Lt | -0.127 | 0.085 | -0.177 |
|  | 0.468 | 0.633 | 0.310 |
| granule cell layer of dentate gyrus _head_Rt | -0.163 | 0.025 | -0.156 |
|  | 0.349 | 0.890 | 0.371 |
| hippocampus-amygdala-transition _Lt | 0.142 | 0.250 | -0.012 |
|  | 0.417 | 0.154 | 0.946 |
| hippocampus-amygdala-transition _Rt | -0.176 | 0.259 | -0.256 |
|  | 0.313 | 0.139 | 0.137 |
| hippocampal_fissure_Lt | 0.209 | 0.005 | 0.079 |
|  | 0.228 | 0.979 | 0.652 |
| hippocampal_fissure_Rt | 0.241 | 0.123 | 0.039 |
|  | 0.163 | 0.489 | 0.822 |
| Hippocampal_tail_Lt | 0.103 | 0.060 | 0.034 |
|  | 0.554 | 0.735 | 0.847 |
| Hippocampal_tail_Rt | 0.163 | 0.004 | 0.063 |
|  | 0.350 | 0.981 | 0.721 |
| Lateral_nucleus_Lt | 0.069 | 0.268 | -0.117 |
|  | 0.694 | 0.126 | 0.505 |
| Lateral_nucleus_Rt | -0.042 | 0.276 | -0.213 |
|  | 0.810 | 0.114 | 0.220 |
| Medial_nucleus_Lt | 0.079 | 0.369 | -0.221 |
|  | 0.650 | 0.032 | 0.201 |
| Medial_nucleus_Rt | -0.048 | 0.166 | -0.221 |
|  | 0.783 | 0.349 | 0.203 |
| molecular_layer_HP_body_Lt | -0.027 | 0.048 | -0.075 |
|  | 0.880 | 0.787 | 0.669 |
| molecular_layer_HP_body_Rt | -0.174 | 0.031 | -0.226 |
|  | 0.316 | 0.863 | 0.192 |
| molecular_layer_HP_head_Lt | -0.014 | 0.187 | -0.167 |
|  | 0.936 | 0.290 | 0.336 |
| molecular_layer_HP_head_Rt | -0.124 | 0.191 | -0.260 |
|  | 0.476 | 0.280 | 0.131 |
| Paralaminar_nucleus_Lt | -0.099 | 0.286 | -0.237 |
|  | 0.571 | 0.101 | 0.170 |
| Paralaminar_nucleus_Rt | -0.029 | 0.397 | -0.253 |
|  | 0.868 | 0.020 | 0.143 |
| parasubiculum_Lt | -0.190 | 0.030 | -0.133 |
|  | 0.275 | 0.866 | 0.446 |
| parasubiculum_Rt | -0.250 | 0.256 | -0.260 |
|  | 0.147 | 0.144 | 0.131 |
| presubiculum_body_Lt | -0.270 | -0.077 | -0.228 |
|  | 0.116 | 0.664 | 0.187 |
| presubiculum_body_Rt | 0.010 | 0.213 | -0.129 |
|  | 0.953 | 0.227 | 0.461 |
| presubiculum_head_Lt | 0.002 | 0.209 | -0.139 |
|  | 0.991 | 0.237 | 0.425 |
| presubiculum_head_Rt | -0.104 | 0.324 | -0.310 |
|  | 0.552 | 0.061 | 0.070 |
| subiculum_body_Lt | -0.068 | -0.028 | -0.076 |
|  | 0.698 | 0.875 | 0.662 |
| subiculum_body_Rt | 0.112 | 0.104 | 0.017 |
|  | 0.521 | 0.558 | 0.924 |
| subiculum_head_Lt | 0.135 | 0.288 | -0.138 |
|  | 0.439 | 0.098 | 0.430 |
| subiculum_head_Rt | -0.043 | 0.352 | -0.313 |
|  | 0.807 | 0.041 | 0.067 |
| Whole_hippocampal_body_Lt | -0.072 | -0.003 | -0.085 |
|  | 0.679 | 0.986 | 0.629 |
| Whole_hippocampal_body_Rt | -0.105 | 0.093 | -0.178 |
|  | 0.549 | 0.601 | 0.305 |
| Whole_hippocampal_head_Lt | -0.023 | 0.186 | -0.165 |
|  | 0.895 | 0.293 | 0.342 |
| Whole_hippocampal_head_Rt | -0.143 | 0.213 | -0.271 |
|  | 0.411 | 0.227 | 0.115 |

The number above represents the correlation coefficient value, and the number below reveals the *p*-value.
